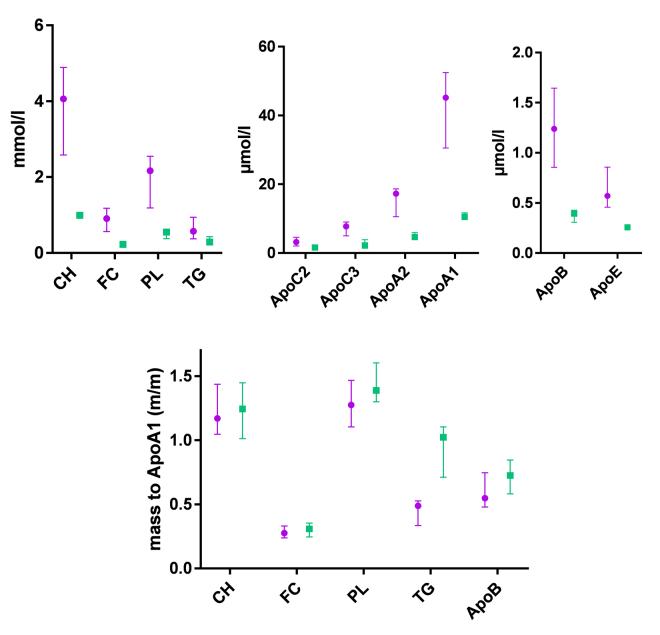# Modeling the postprandial CETP-mediated lipid redistribution between chylomicrons, LDL and HDL

**DOI:** 10.1016/j.jlr.2025.100847

**Published:** 2025-06-23

**Authors:** Martin Jansen, Christine Contini, Michael M. Hoffmann, Gerhard Puetz

**Affiliations:** 1 Institute of Clinical Chemistry and Laboratory Medicine, Medical Centre-University of Freiburg, Freiburg im Breisgau, Germany; 2 Faculty of Medicine, University of Freiburg, Freiburg im Breisgau, Germany

**Keywords:** lipid transfer protein, chylomicrons, lipoproteins/metabolism, LDL/metabolism, triacylglycerol, VLDL

## Abstract

Impaired triglyceride (TG) metabolism is associated with metabolic diseases. Non-steady state dynamics make studying postprandial lipid metabolism challenging. We already introduced a mathematical model to estimate cholesteryl ester transfer protein (CETP)-mediated TG net flux in the fasting state. Here, we expand this model to chylomicrons (CMs) and the dynamics of postprandial lipemia. Blood samples of normolipidemic, hypertriglyceridemic, and hyperchylomicronemic volunteers were drawn at fasting and postprandial state. We separated lipoprotein classes via classical sequential ultracentrifugation. To address CMs, we developed a novel method based on Airfuge® ultracentrifugation. We studied postprandial changes of lipoproteins and their components. CETP-mediated TG redistribution was modeled based on the surface and composition data of respective lipoprotein fractions and validated by corresponding measured values. Our model estimated CETP-mediated TG flux in the fasting and postprandial state with high accuracy. Even in the postprandial condition, TG net flux to LDL/HDL is dominated by VLDL. Separating CM from VLDL and modeling both fractions instead of just using the combined CM + VLDL fraction did only improve the model’s accuracy slightly (by less than 7%). The proportion of ApoC3 redistributed from HDL to VLDL in postprandial lipemia is highly correlated with the change of ApoA1 in HDL2b. Our basic model is able to estimate TG redistribution via CETP among lipoproteins in postprandial lipemia of healthy and hypertriglyceridemic subjects. An additional separation of VLDL and CM is not strictly necessary to model postprandial TG flux. Our model makes postprandial lipoprotein metabolism more tangible and may help to study lipoprotein-associated pathologies.

Understanding the metabolism of lipoproteins is crucial, as it is associated to diseases with devastating consequences in Western societies. Especially an impaired cholesterol (1) or triglyceride (TG) metabolism (2) is of central importance, as both are associated to atherosclerosis and the metabolic syndrome, respectively. Independent of traditional cardiac risk factors, cardiovascular risk is associated with the nonfasting TG levels (3) and with chylomicron remnants (CMRs) (4, 5).

In contrast to cholesterol’s level, plasma TG level shows a huge variability throughout the day, as ingestion may lead to postprandial lipemia—a non-steady state and dynamic condition mainly characterized by the temporary increase of CMs and VLDLs. After digestion, plasma TG increases and peaks around 4 h (6, 7). The metabolism of VLDL is impaired during postprandial lipemia, as CM competes with VLDL for hydrolysis, and insulin signaling slows down VLDL production (8, 9, 10). These non-steady state condition causes additional complexity to the TG metabolism compared to the cholesterol metabolism, which makes it more challenging to study the TG metabolism in lipoproteins.

On average, CMs consist of about 90% TG in the postprandial state and VLDLs consist of 54% TG by mass (11). CMs are transformed into CMR by hydrolysis of TG, and these CMRs are finally cleared from circulation. Additional factors like the apolipoproteins C-II (ApoC2), C-III (ApoC3), E (ApoE), and Angiopoietin-like 4 play an important role in the hydrolysis of TG. Data on the dynamics of redistribution of ApoC3 and ApoC2 between lipoproteins are sparse. ApoC2 acts as a cofactor for lipoprotein lipases (12), whereas apoC3 is associated with inhibitory roles in TG catabolism and redistribution (13). ApoC2 and ApoC3 are mainly located in VLDL and large HDL (14). In postprandial lipemia, they redistribute from HDL to VLDL (15). While nascent CM accept ApoC2 from HDL particles, CMR donates ApoC2 to HDL, correspondingly. Hence, the capability of HDL to provide a pool of regulatory apolipoproteins for CM and VLDL may affect proatherogenic or antiatherogenic properties of HDL. Human CMs are characterized unambiguously by apolipoprotein B (ApoB) isoform ApoB-48. Compared to the ApoB concentration in VLDL, the concentration of ApoB-48 (and respectively the amount of CM particles) is very low, not only in the fasting but in the postprandial state of normolipidemics as well (16).

Compared to normolipidemics, hypertriglyceridemics exhibit a higher concentration of CMRs caused by a delayed catabolism (17).

Familial hyperchylomicronemia syndrome (FCS) is characterized by elevated TG values caused by an impaired catabolism of CM for example due to genetic defects in lipoprotein lipase or ApoC2.

Cholesteryl ester transfer protein (CETP) distributes TGs and cholesteryl esters (CEs) among lipoproteins in blood. CETP exchange depends on factors like the availability of lipids in the donor surface (18), CETP’s substrate preference (CE or TG) (19) and the lipid composition of donor and acceptor lipoproteins (20). We and others assume that CETP exchange resembles carrier-mediated diffusion of TG and CE amongst all lipoproteins (21, 22). In general, CETP provides a transport of TG from the TG-rich lipoproteins (TRLs) CM and VLDL to the TG-poor lipoproteins LDL and HDL and a CE transport in the converse direction. There is evidence that CETP plays a proatherogenic role (23). However, pharmaceutical inhibition of CETP as therapeutic intervention did not meet ones expectations, yet (24).

Based on the assumption that CETP mediates a diffusion-like distribution of TG among lipoproteins, we introduced a novel method to estimate CETP-mediated lipid flux among lipoproteins from a single blood sample (22). This model is based on basic lipoprotein composition data and measured CETP-mediated TG redistribution amongst lipoproteins and estimated the CETP-mediated TG flux with high precision under fasting steady state condition. Lipoprotein composition data were obtained by isolation of TRL (VLDL + CM + CMR), IDL, LDL, and HDL by classical sequential ultracentrifugation (UC) and subsequently measurements of lipids and apolipoproteins.

Measurement of CM is rather challenging and—besides the mere qualitative “refrigerator test”—unusual in patient care. Due to overlapping densities, separating CM and CMR from VLDL via UC is only partially possible. Immunoaffinity methods are able to separate CM and CMR from VLDL (25) but are very elaborate and costly.

In this work, we present a new way to quantify CM and use these data to characterize the lipid profile in normolipidemic, hypertriglyceridemia (HTG), and FCS persons at fasting and postprandial state in detail. Further, we measured TG redistribution among all lipoprotein classes including CM. Based on these data, we tested, whether the established CETP model is valid for the postprandial state as well. Furthermore, we tested whether separate modeling of CM and VLDL yields additional accuracy or can be simplified by a combined TRL fraction that is much easier to measure. Additionally, we analyze the redistribution of apolipoproteins after food intake, as corresponding data are still sparse in the literature.

## Materials and methods

### Study protocol

#### Subjects

The study was approved by the Freiburg Ethics Committee (UTN: U1111-1265-0934, DRKS00024609, DRKS00035158) and conducted in accordance with the Declaration of Helsinki. Fifteen healthy, 2 HTGs, and 2 FCSs (both exhibit a defect in lipoprotein lipase) participants were recruited and gave informed consent. Blood samples were taken in the morning at a fasting state and except for the FCS participants 3–4 h after a fat-rich meal (breakfast or lunch) chosen by the participants.

#### Measurement of lipids, apolipoproteins, and CETP-mediated TG redistribution

Except for the CM separation, the experimental procedures were described in detail previously (26). Briefly, plasma samples were divided into two aliquots, one was kept at 4°C and one was kept at 37°C for 1 h before separation. The measured delta of TG in respective lipoprotein fractions was considered as CETP-mediated redistribution. Plasma was separated into VLDL, IDL, LDL, HDL, lipid-deficient serum as well as into six LDL (LDL1–LDL6) and three HDL (HDL2b, HDL2a, and HDL3) subfractions by density gradient UC. TG, free cholesterol (FC), phospholipid (PL), and total cholesterol were determined enzymatically with PAP-Methods (DiaSys; Diagnostic Systems GmbH, Holzheim, Germany) using a Beckman AU680 autoanalyzer. The difference between the total cholesterol and FC was interpreted as CE. Turbidimetric methods were used to determine ApoB, A1 (ApoA1) (DiaSys; Diagnostic Systems GmbH) and A2 (ApoA2), C2 (ApoC2), C3 (ApoC3), and E (ApoE) (Kamiya Biomedical Company, Tukwila, WA). The immunoturbidimetric ApoB test determines both ApoB-100 and ApoB-48. We used the molar weight of ApoB-48 in the CM fraction and the molar weight of ApoB-100 elsewhere. The CETP-mediated redistribution was determined by considering the delta of TG in VLDL, IDL, LDL, and HDL between the two aliquots. FC, CE, PL, and TG were measured in all fractions.

### CM measurement

**Fig. 1:**
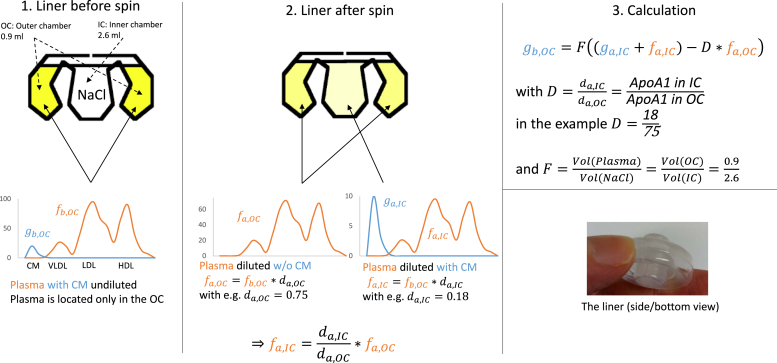
Calculation of CM-associated components. *g* and *f* denote the concentration of a lipid or an polipoprotein in CMs or plasma without CMs (illustrated as exemplary chromatography), respectively, either before (b) or after (a) spin in the inner chamber (IC) or outer chamber (OC) of the liner. The dilution factors $d_{a,OC}$ and $d_{a,IC}$ are estimated for each sample via ApoA1 concentration.

Figure 1 gives an overview of our approach to estimate concentrations of CM and its components. We used an Airfuge® Ultracentrifuge (Beckman Instruments, Palo Alto, CA) equipped with an ACR-90 CM rotor with 3.5 ml liners, which consists of an outer and inner chamber of 2.6 ml and 0.9 ml volume, respectively. Contrary to the standard protocol, we filled the inner chamber of the liner with 154 mM NaCl solution, whereas the outer chamber was filled with the plasma sample. During the spin, a small channel forms on top of the chambers, allowing a flux of floating material and a certain amount of plasma from the outer to the inner chamber. After a 10 min spin at 90,000 rpm at room temperature, we collected material of the inner and outer chambers. Subsequently, apolipoproteins and lipids were determined. After centrifugation, low-density CMs are located exclusively in the inner chamber (instruction manual “ACR-90 rotor”; Beckman Coulter), thus the remaining plasma in the outer chamber is CM free (Fig. 1). Unfortunately, the concentration of lipid components measured in the inner chamber $c_{in}\left(X\right)$ consists of CM-associated lipids $c^{chylo}\left(X\right)$ and a certain amount of “contaminating” lipids $c_{in}^{plasma}\left(X\right)$ from transferred plasma during centrifugation. To calculate the concentration of CM-associated lipids, Equation 1 (which equals Equation 6 in the supplement) was used (see Fig. 1 and supplement for details):

$$g_{b,OC}=F\left(\left(g_{a,IC}+f_{a,IC}\right)-D*f_{a,OC}\right),\left(\text{eq}.\;1\right)$$

With:

• $g_{b,OC}$ as the concentration of CM-associated component *X* in plasma
• $F=\frac{0.9}{2.6}$ coefficient for the two-chamber volumes
• $D=\frac{c_{in}\left(ApoA1\right)}{c_{out}\left(ApoA1\right)}$ the coefficient for non-CM-associated ApoA1 concentration
• $g_{a,IC}+f_{a,IC}=c_{in}\left(X\right)$, where $g_{a,IC}+f_{a,IC}$ denotes the sum of *X*’s concentration in CM and non-CMs after spin in the inner chamber
• $f_{a,OC}=c_{out}\left(X\right)$, where $f_{a,OC}$ denotes *X*’s concentration in the outer chamber after spin.

### Modeling impact of CMs

The CETP-mediated net flux of TG to all lipoprotein classes was calculated as described in our previous work using a single TRL fraction (22). This model was validated for the fasting state. By standard density gradient UC, a TRL fraction is obtained, which mainly consists of VLDL, CM, and CMR. In the fasting state, residual CM and CMR are neglected, and the TRL fraction is generally denoted as VLDL fraction. In the postprandial state, the amount of CM and CMR increases, raising the question whether the use of a single TRL fraction is accurate to describe TG flux in the postprandial state.

Thus, the TG flux model has to be validated for postprandial conditions.

By estimating CM as described above, we divided the TRL fraction obtained by standard density gradient UC (VLDL, CM, and CMR) into a CM fraction and fraction containing VLDL and CMR (notation: VLDL + CMR). Note that (as pointed out in the discussion) traces of CMRs may also be located in the CM fraction. Hence, TRL can be interpreted in this article as the union of the fractions CM and VLDL + CMR. To model postprandial lipoprotein metabolism, we replaced the TRL fraction by those two fractions in the calculation of net fluxes for the postprandial state. Note that the calculated surfaces as described in our previous work (22) of all lipoprotein fractions are necessary for the model construction.

## Statistics

All statistics were performed by SPSS, version 21.0 (IBM SPSS Statistics, IBM Corporation, Chicago, IL). Data are presented in quartiles. Pairwise differences were studied with Wilcoxon signed-rank test. Correlations were calculated by Spearman’s correlation.

## Results

### Estimation of CM amount and composition via Airfuge

In order to obtain CM compositional data, plasma was centrifuged in a special Airfuge ACR-90 rotor liner described in the Method part. We measured lipids and apolipoproteins in both chambers (Supplemental Fig. S2) and normed the results to ApoA1. In general, the concentration of all components was significantly higher in the outer chamber, indicating that only a small plasma fraction crosses from the outer to the inner liner during the spin. Nevertheless the ratios TG/ApoA1, but also PL/ApoA1 and ApoB/ApoA1, were higher in the inner chamber, which suggest that CMs are concentrated in the inner chamber as expected. In contrast, the ratio of essentially non-CM components CH and familial combined hyperlipidemia to ApoA1 is almost equal among both chambers.

**Fig. 2:**
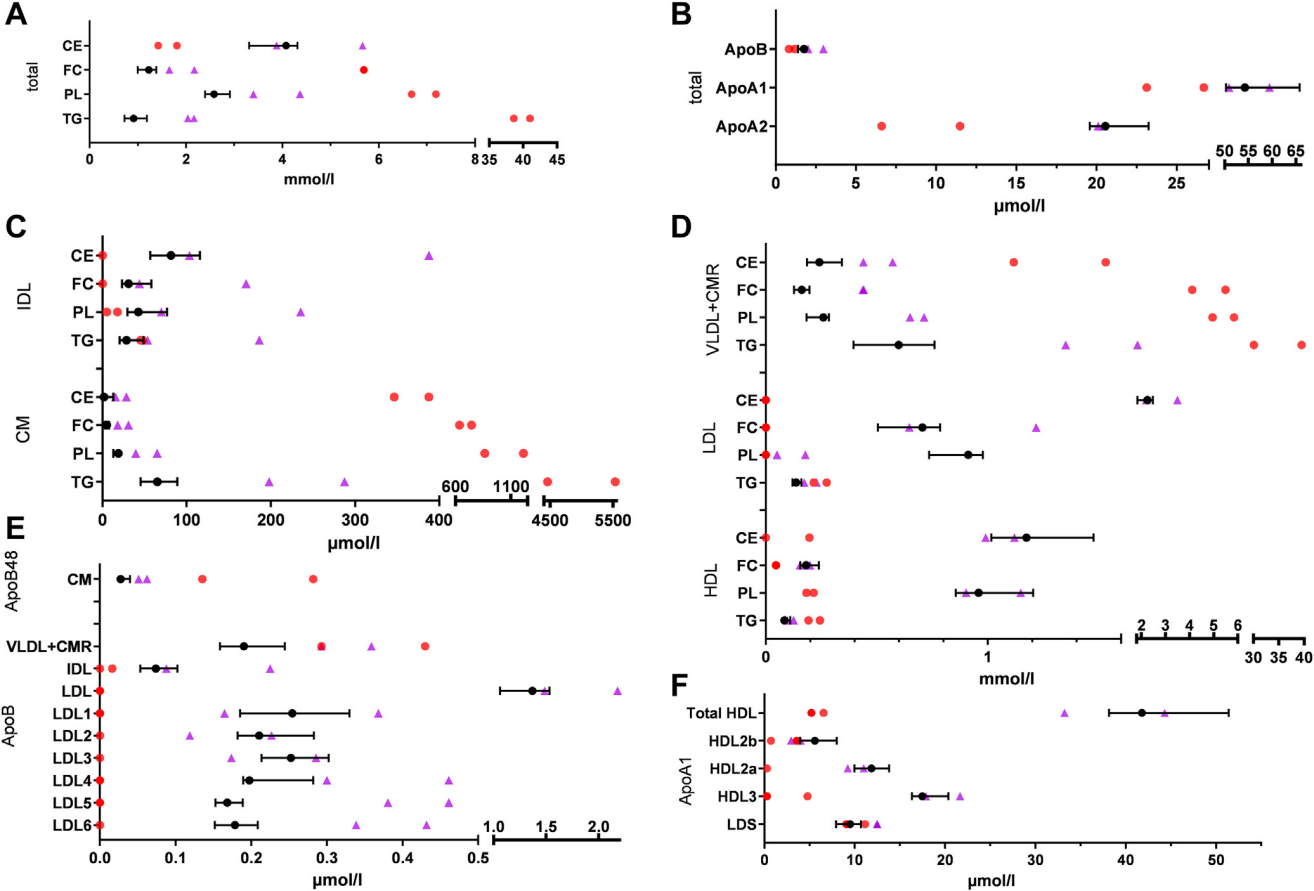
Concentrations of lipids and apolipoproteins at fasting state. Lipids total (A), apolipoproteins total (B), lipids from CM and IDL (C), lipids in VLDL + CMR, LDL, and HDL (D), ApoB-100 and ApoB-48 lipoprotein fractions and LDL subfractions (E), and ApoA1 in HDL, LDS, and HDL subfractions (F). Black circle with whiskers: quartiles of normolipidemics (n = 12), purple triangles: hypertriglyceridemics (n = 2), and red circles: hyperchylomicronemia (n = 2). LDS, lipid-deficient serum.

As expected, the two FCS samples displayed very high TG levels around 40 mmol/l (Fig. 2). These levels led to difficulties in lipoprotein separation and the subsequent measurements. So UC (either in the Airfuge or in the Beckman rotor) led to solid white clots and hence to loss of material. For the subsequent isolated IDL, LDL, and HDL, additional dilution steps were necessary. These impairments led to a loss of precision in the measurement of lipids and apolipoproteins for FCS samples.

### Baseline data

Of the 15 normolipidemic participants, we excluded three participants as their total TG did not distinctly (>20%) change in postprandial lipemia. In total, data of seven normolipidemic, two HTG and two FCS males, and five normolipidemic females were used for our evaluation (Fig. 2).

The compositions of lipoprotein fractions in the fasting state are given in Figure 2. In the fasting state, the level of ApoB in the CM fraction in normolipidemics amounted about 27.5 nmol/l, which is close to the detection limit of 18 nmol/l. Most abundant components of CM were TG and PL, as expected. In normolipidemics, ApoB concentration of CM is about 1.6% of total ApoB and about 13% of combined TRL. Similar in normolipidemics, CM TG accounted for about 7% of total TG and 10% of TRL TG. LDL’s subfraction profile, total TG level, and LDL cholesterol of the normolipidemics were all in a nonpathologic range (Fig. 2).

Both HTG participants were slightly above 2 mmol/l TGs, whereas the FCS participants had total TGs above 35 mmol/l. ApoB and lipid levels in CM were clearly higher in both FCS samples compared to HTG samples. The ApoB concentrations of CM in HTGs are about 10% of TRL ApoB and 1% of total ApoB. In FCS, they are about 20% and 8%, respectively.

### Change after food intake in normolipidemics

**Fig. 3:**
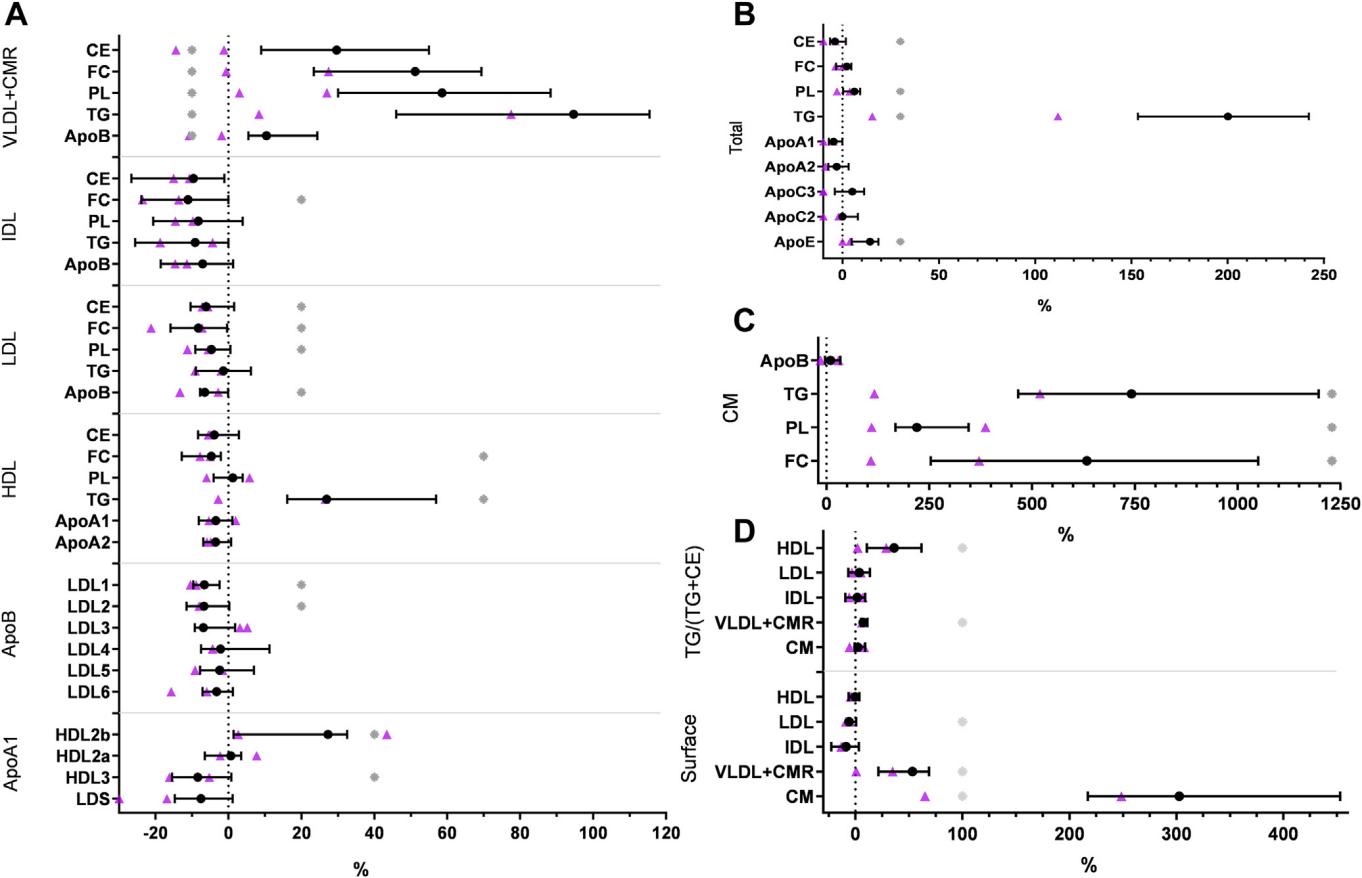
Percentage change between fasting and postprandial (3–4 h after ingestion) state in lipids and apolipoproteins of the lipoprotein fractions VLDL, IDL, LDL + subfractions, HDL + subfractions (A), total plasma (B), CM (C), and calculated parameters, which are important for modeling redistribution of TG via CETP (D). Black circle with whiskers: quartiles of normolipidemics (n = 12) Wilcoxon signed-rank test, purple triangles: hypertriglyceridemics (n = 2).

**Fig. 4:**
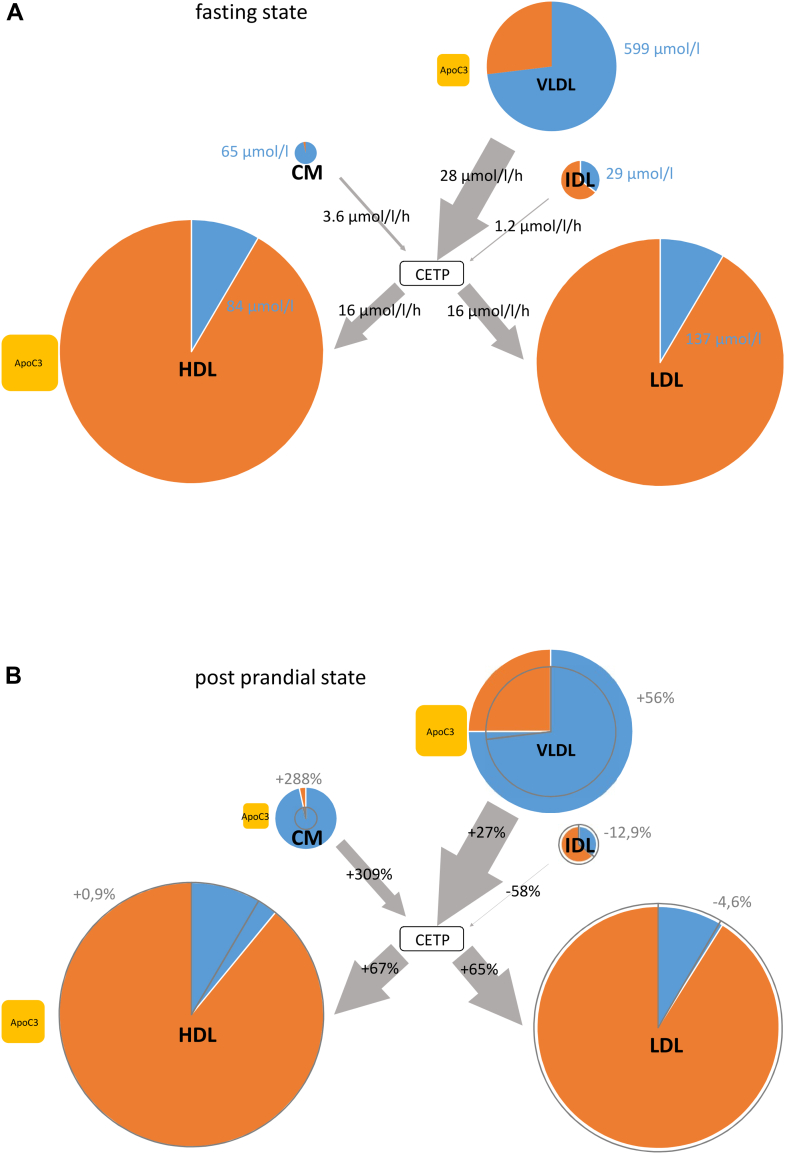
Summary: The effect of postprandial state on CETP-mediated TG-flux and lipoprotein fractions. CETP-mediated TG net fluxes at fasting (A) and postprandial state (B) in normolipidemics (n = 12). The surface of the circles (lipoprotein fractions), rectangles (ApoC3), and the thickness of the arrows are proportional to the surface of the fractions, the concentration of ApoC3 and the TG net flux, respectively. The proportion of TG/(CE + TG) and CE/(CE + TG) in the fractions is displayed blue and orange, respectively. The CETP-mediated TG net fluxes and the TG concentration of the fractions (blue) are displayed (A). The change in surface and the CE-TG proportion at postprandial state is displayed in (B) in gray. Changes in CM, VLDL, and LDL surface are significant. CMRs are included in the VLDL fraction.

**Supplemental Figure S1:**
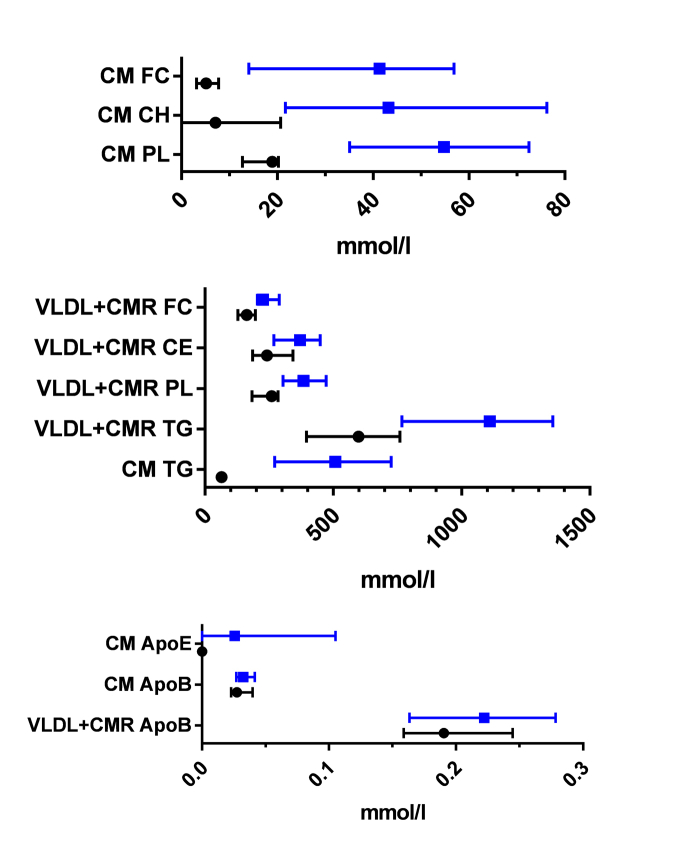


Due to the low number of HTG and FCS cases, the following statistic results refer only to the normolipidemic cases: Figure 3 displays the percentage change of apolipoproteins and lipids in postprandial lipemia. Total concentrations of the separated VLDL + CMR and CM fractions are given in Supplemental Figure S1. The concentration of lipids in the CM fraction increased significantly and considerably (PL, FC, and TG by factor 4, 7, and 8, respectively), whereas ApoB increased only slightly. Interestingly, ApoB in VLDL + CMR increased by a higher extent than CM ApoB in the postprandial state. ApoB decreased significantly in the two most buoyant LDL subfractions LDL1 and LDL2, which causes total LDL’s surface reduction (Fig. 4). ApoA1 in HDL3 decreased and ApoA1 in HDL2b increased significantly. However, total HDL surface does not change significantly. While all components in VLDL + CMR increased significantly, all LDL components but TG decreased significantly. HDL TG increased, whereas HDL CE decreased significantly.

**Fig. 5:**
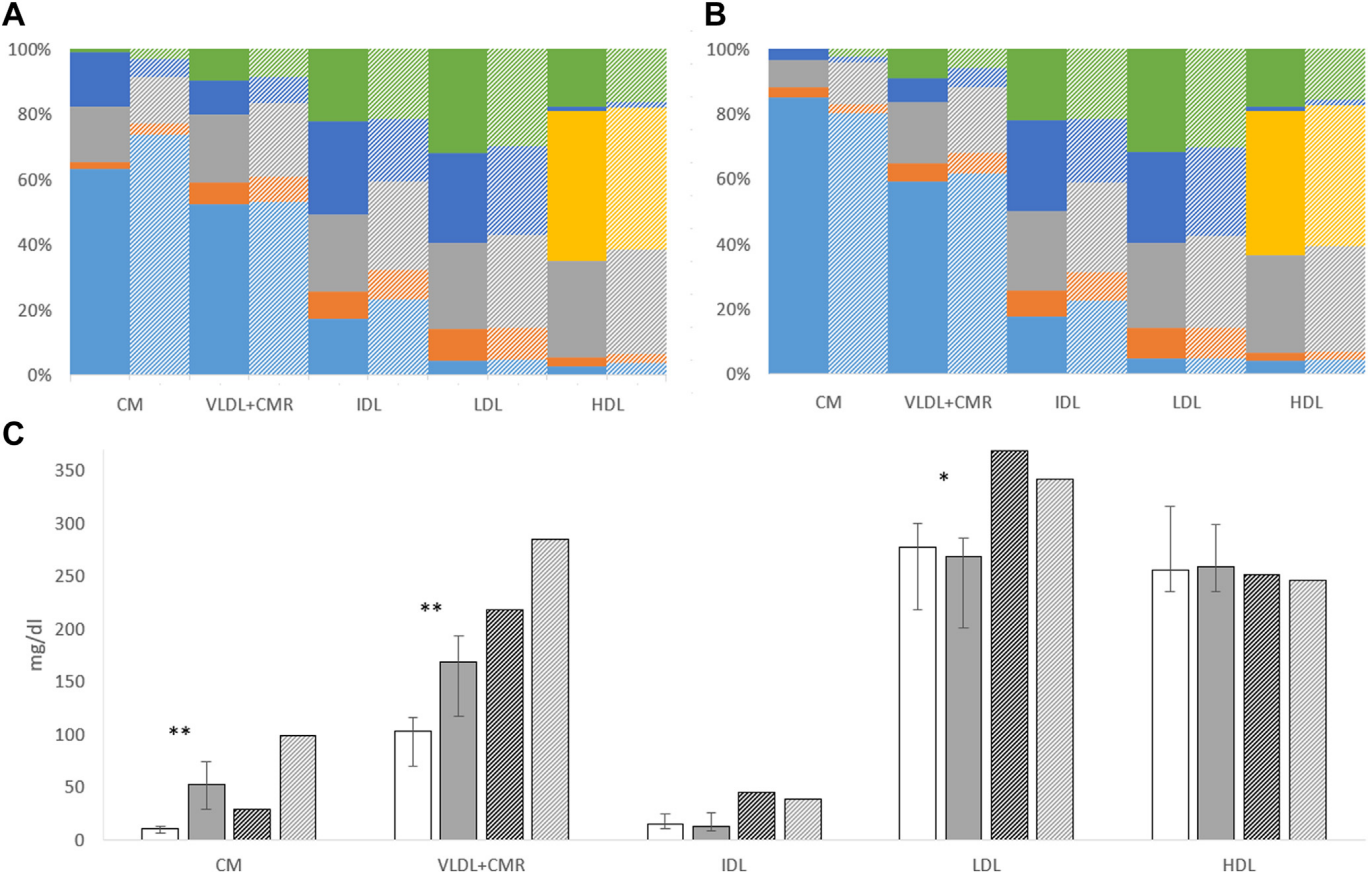
Lipoprotein composition (median) in fasting (A) and postprandial (B) state (wt/wt) in normolipidemics (solid) and hyperlipidemics (n = 2, dashed). Light blue = TG, orange = FC, gray = PL, dark blue = ApoB, yellow = ApoA1, and green = CE. Concentration (median and quartiles) of these components combined in fasting (white solid bars) and postprandial (gray solid bars) state (C) in normolipidemics (solid) (∗<0.05, ∗∗<0.01, Wilcoxon signed-rank test, n = 12) and hyperlipidemics (n = 2, fasting: black dashed bars, postprandial: gray solid bars).

Figure 5 displays the lipid and apolipoprotein composition of CM, VLDL + CMR, IDL, LDL, and HDL in fasting and postprandial state. Especially, CM’s composition changed toward a higher TG and a lower protein proportion.

**Fig. 6:**
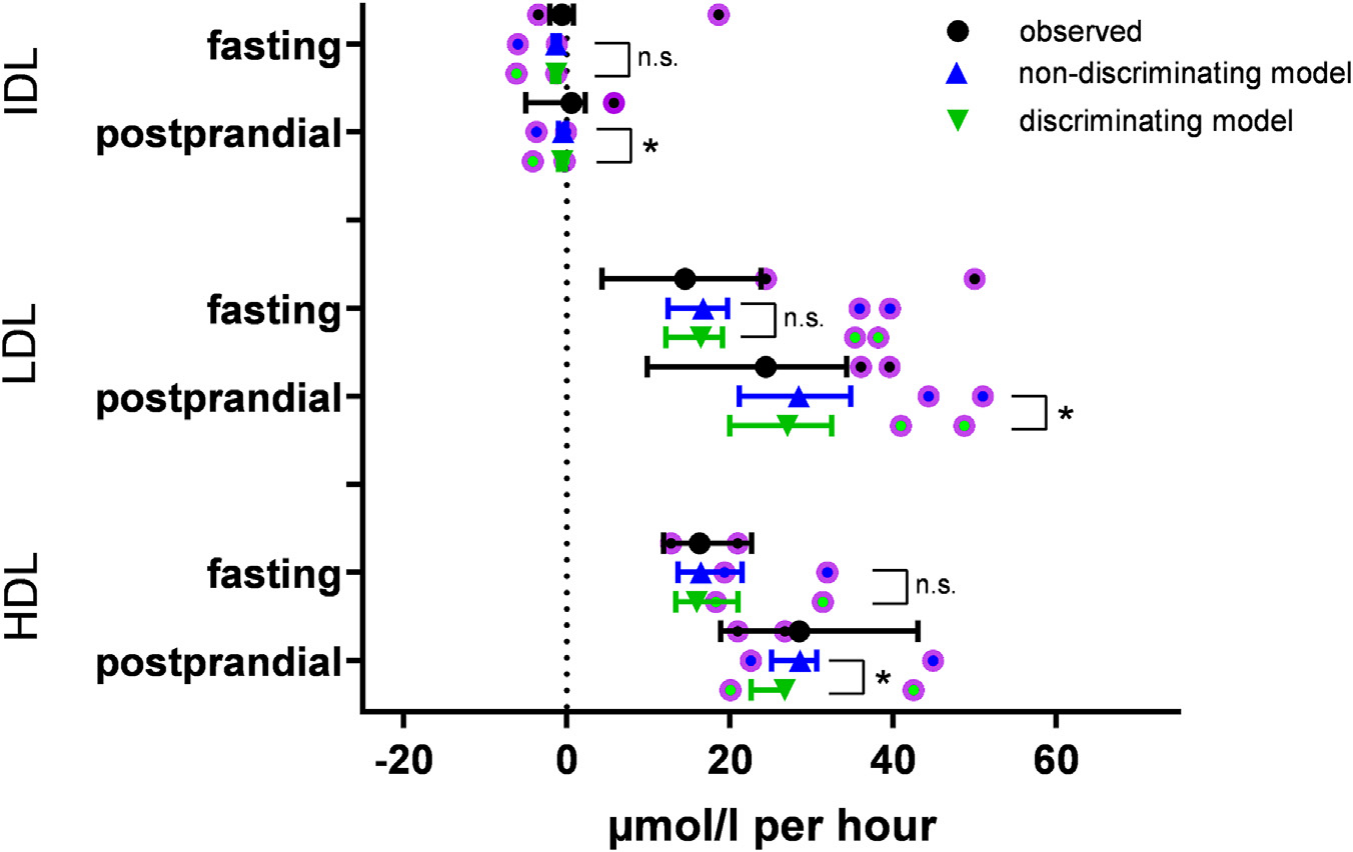
Comparison between observed = black and modeled (either “not discriminating model” = blue or “discriminating model” = green) TG net fluxes in LDL, IDL, and HDL per hour (circle with whiskers: quartiles of n = 12 normolipidemics, circles with purple border: hypertriglyceridemics [n = 2]). Fluxes were modeled in two ways: Using data, either in which CM and VLDL are merged (nondiscriminating model) or in which both fractions are used (discriminating model). Significant differences between both models in the normolipidemic case are displayed (Wilcoxon signed-rank test). Observed data are derived by subtracting TG in the corresponding fractions from samples stored either for 1 h at 37°C (before separation and measurement) or not.

Considering TG in TRL, 9.8% and 31.4% are located in CM at fasting state and postprandial lipemia, respectively. The CETP-mediated net flux of TG into LDL and HDL as well as TG out of VLDL + CMR doubles in the postprandial state (Fig. 6, black circles).

#### Change after food intake in non-normolipidemics

The percentual change of total TG in both HTG volunteers clearly differed (+15% and +112%). The concentration of lipids in the CM fraction increased distinctly. ApoB in LDL and ApoA1 in HDL and its subfraction showed the same tendencies as in the normolipidemic cases.

#### Change of model parameters in normolipidemics

The ratio TG/(TG + CE) and the surface of the lipoprotein fractions were calculated as described previously (22).

Postprandial, the TG/(TG + CE) ratio increased significantly in HDL and VLDL. Our model predicts that CETP-mediated net TG flux into HDL and LDL per hour at fasting state amounts to 22% of HDL’s and 11% of LDL’s total TG content (Fig. 4).

While the surface of the LDL fraction decreased, the surface of all TRLs increased significantly. Compared to VLDL and CMR, the larger CM particles were 40% more likely to mediate a net flux of TG to other fractions in both the fasting state and postprandial state. Even though the relative surface increase in the CM fraction was five times higher than in the VLDL + CMR, the absolute surface area in VLDL + CMR exceeded the CM surface area even in the postprandial state significantly by factor 2.4.

#### Change of model parameters in non-normolipidemics

As in the normolipidemic case, the TG/(TG + CE) ratio increased in HDL and VLDL, the surface of all TRLs increased, and LDL surface decreased.

#### FCS samples

In the FCS samples, the composition and concentration of the lipoprotein classes were severely distorted and differed clearly from the normolipidemic samples, considering the composition of VLDL + CMRs and CM in FCS are both comparable with normolipidemic CM and carry nearly the total mass of lipids and apolipoproteins. LDL and HDL have lipid compositions, which differ strongly from the normo- and “normal” HTG cases and are clearly lower concentrated.

### Model with and without discrimination of CMs in normolipidemics

The so called “VLDL” fraction from classical UC is strictly speaking the combined fraction of VLDL, CM, and CMR, named TRL in this article to avoid confusion. To test whether a separation of this TRL into CM and VLDL + CMR is necessary for accurate modeling, we displayed the results of the “nondiscriminating model” (as described in Ref. (22)), which does not discriminate between both fractions, and the results of the “discriminating model,” which does discriminate TRL into CM and VLDL + CMR. Comparing both configurations (Fig. 6), there are no significant differences between all lipoproteins in the fasting situation. In postprandial lipemia, TG enrichment in LDL and HDL is lower and TG loss in IDL is higher in the discriminating model (*P* = 0.002, 0.002, and 0.002). However, the difference in calculated TG net fluxes is small, 7%, 7%, and 17%, respectively. As the modeled values between both configurations are highly correlating for each fraction, it is not surprising that even small differences are highly significant.

### Model with and without discrimination of CMs in non-normolipidemics

The separation of the classic UC fraction TRL into CM and VLDL + CMR led to lower predicted TG enrichment in LDL (4%) and HDL (5%) values in both cases. Compared to the postprandial situation in the normolipidemic samples, it was however clearly lower (8% in LDL and 11% in HDL, respectively).

In the FCS situation, the corresponding effect was <2% in LDL and HDL. The model predicted nearly no TG net flux into LDL and a low TG net flux (about 10 μmol/l per hour) from TRL to HDL. Though our measured TG net flux data were not as accurate in the FCS samples as in other samples, as discussed earlier, respective prediction seems reasonable as the CETP redistribution might nearly have reached its steady state.

### Change of ApoC3 and ApoC2

**Fig. 7:**
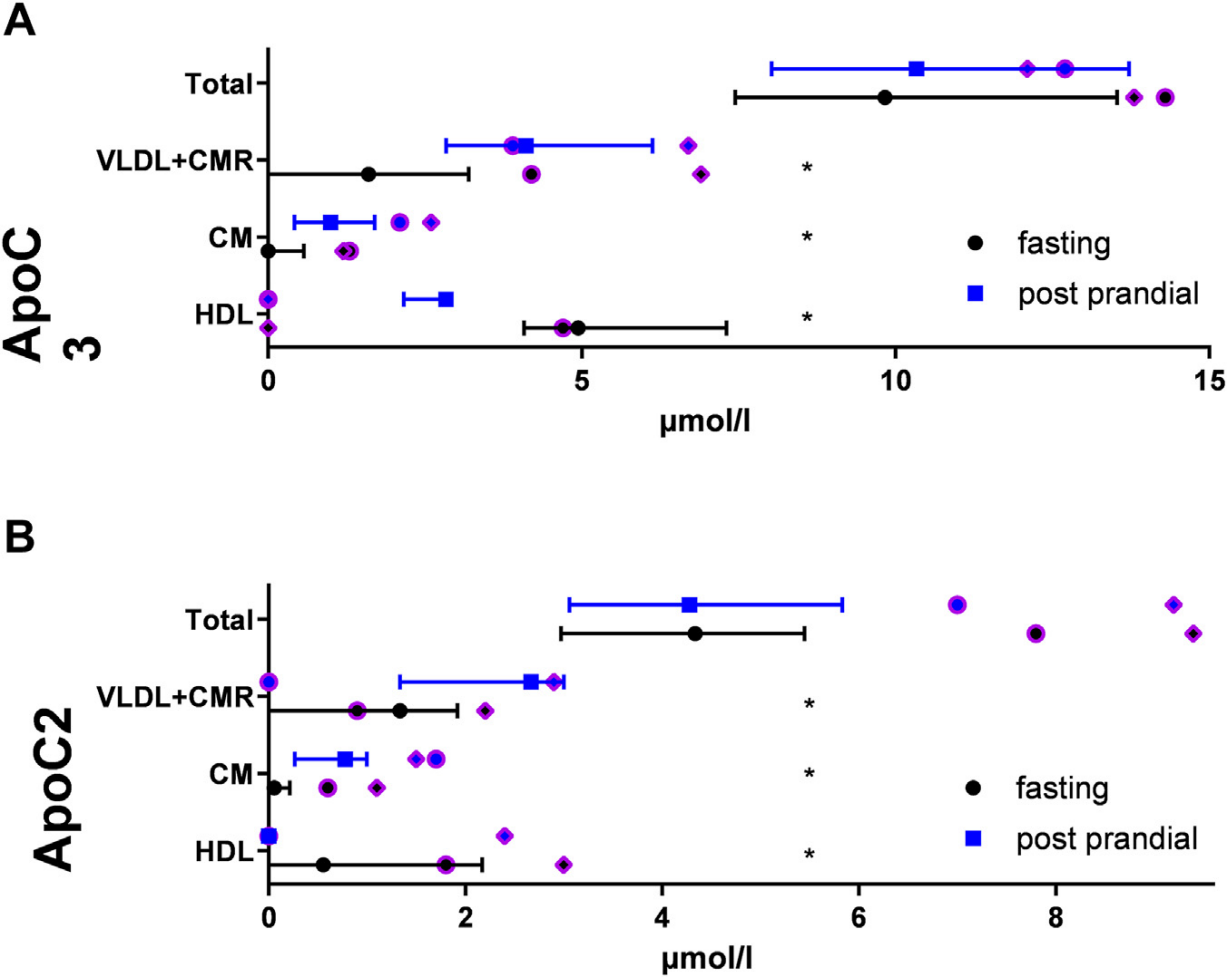
Concentration of ApoC3 (A) and ApoC2 (B) in total, CM, VLDL, and HDL at fasting (black) and postprandial state (blue) in normolipidemics (n = 12) and hyperlipidemics (n = 2, purple borders).

Figure 7 displays the redistribution of ApoC2 and ApoC3 after food intake. In the fasting state, ApoC3 is mainly located in the HDL fraction. In postprandial lipemia, it is located in VLDL + CMR and to a minor part in CM. In normolipidemics, the total mass of ApoC2 and ApoC3 does not change significantly.

We checked in an additional analysis in normolipidemics (Table 1) what factors are correlated to the redistribution of ApoC3 from HDL to TRL in normolipidemics. The highest correlating factor is the change of ApoA1 in HDL2b: The more ApoA1 accumulates in the HDL2b fraction in the postprandial state the more ApoC3 is redistributed to TRL. The ratio HDL surface/(HDL + TRL surface) is also significantly correlated.

**Table 1:** Correlations associated to redistribution of ApoC3 after food intake

Parameter	Rho	*P*
ΔHDL2b ApoA1	−0.874	0.0002
ΔHDL ApoC3	0.832	0.0008
ΔTotal TG	−0.818	0.0011
ΔCM PL	−0.764	0.0062
ΔHDL surface/(HDL + VLDL surface)	0.664	0.0185
ΔVLDL TG	−0.615	0.0332
ΔHDL2a ApoA1	−0.587	0.0446

All parameters, which significantly correlate (Spearman’s rho) to the ratio ΔHDL_ApoC3/(HDL_ApoC3 + VLDL_ApoC3). Δ denotes the change between fasting and postprandial state (normolipidemics, n = 12).

We did not perform a corresponding analysis with ApoC2, as it was close to detection limit in the TRL and HDL fractions in many samples.

## Discussion

TG metabolism is well known to play a distinctive role in several metabolic diseases, for example, metabolic syndrome, affecting large population cohorts. While fasting TG levels are easy to measure, they reflect only a small part of TG metabolism, and non-steady state kinetics of postprandial TG dynamics make the measurement of TG metabolism difficult. Postprandial dynamics primarily involve CM, but knowledge of postprandial metabolic parameters is hampered by analytical difficulties. Although CM and VLDL metabolic origin and biochemical pathways differ strongly, their phenotype is similar and separation of CM from VLDL is not straightforward and elaborate. ApoB-48 is the only factor to distinguish unambiguously between CM and VLDL (27, 28). There are elaborate methods based on UC to isolate CM (29), in which CMs are identified as fraction with Svedberg flotation rate S_f_ > 400.

### Measurement of CMs

We here present a new method to estimate CM concentration and composition, which is based on an established method for CM clearance of plasma (30). In this method, CMs are centrifuged in a rotor that has two chambers, connected by a channel on top of the chambers (which is only open during centrifugation). Low-density CM redistribute from the outer to the inner chamber, resulting in CM-free plasma in the outer chamber and CM enrichment in the inner chamber (Fig. 1). Unfortunately, the CM fraction in the inner chamber is not free of plasma components, which spill over from the outer to the inner chamber during centrifugation.

CM’s lipid and apolipoprotein concentration is calculated by subtracting the corresponding components of non-CM lipoproteins located in the inner chamber from the measured concentrations in the inner chamber. ApoA1 is essentially not CM associated. We assume, that in the volume of plasma spilled into the inner chamber, mutual proportions of each non-CM component to ApoA1 remain similar to the corresponding proportions in the outer chamber (plasma without CM). Thus, non-CM component concentrations in the inner chamber were calculated from the measured ApoA1 concentration in the inner chamber (for more details, see supplement).

The thereby calculated ApoB concentration in CM, and lipid composition in CM and VLDL + CMR (Fig. 5) at fasting and postprandial state, is consistent with corresponding data in the literature (8, 9, 10, 11), validating our approach. Note that our method’s goal is not to prepare isolated CM but to deliver CM composition and concentration data to test our CETP model in postprandial lipemia.

In contrast to CM, we cannot make statements about the distribution of CMR in the outer and inner chambers. However, considering data from the literature (9, 10), we expect CMR to behave like VLDL and to be located in the VLDL fraction. Thus, we assumed that after Airfuge spin, TRLs are separated into CM and a combined VLDL + CMR fraction.

If the TG concentration is very high like in the FCS samples, our method does not work as well as in normolipidemic samples, as TRL in very high concentrations tend to clot during UC. While the Airfuge-spin does only take 10 min, the further separation of non-CM lipoproteins by our classical method takes at least 4 days as several 18 h spins are involved. Though we had no problems with our method in non-FCS sample (total TG = <4.46 mmol/l), in conditions like FCS, where UC leads to clotting of TRLs, our method has its limitations.

In contrast to the classic methods of CM isolation, which include the overlayering of the sample with a sodium chloride density gradient before spin (31, 32) we chose another approach. Both approaches are not able to separate CMs, CMRs, and VLDL exactly as they have overlapping densities. The advantage of the classic method is that the CM fraction is actually isolated. The advantage of our here presented method is that the spin takes only 10 min (not 40 min (31)), and that it is less elaborate, as no further solutions need to be prepared and handled.

### LDL and HDL lipid composition and subfraction phenotype in the postprandial state

Data on postprandial lipoprotein composition fluctuations are sparse. Hence, although this is not the main focus of this article, we give a detailed description on *a*) LDL’s and HDL’s lipid composition, *b*) their subfraction profile, and *c*) the CETP-mediated TG redistribution (Fig. 6). Here, we describe for the first time the CETP-mediated TG flux in the postprandial state.

a. In normolipidemics, we measured a decrease of LDL and HDL-cholesterol (FC + CE) and an increase in HDL-TG in postprandial lipemia. This is in line with the results by Skoczynska *et al*. (33) and Cohn *et al*. (6). Cohn *et al*. (6) reported a significant decrease in LDL-TG. Considering our normolipidemic and HTG cases, there is no significance but a tendency toward this decrease (Fig. 3).
b. We observed a reduction of ApoB in LDL1 and LDL2 and ApoA1 in HDL3, as well as an increase of ApoA1 in HDL2b, that was statistically significant in normolipidemics. This is in line with the studies by Sabaka *et al*. (34), Bellanger *et al*. (35), and Julia *et al*. (36).
c. While the predicted TG net flux into LDL and HDL doubles (Fig. 4) in the normolipidemic samples during postprandial lipemia, only HDL’s TG/(TG + CE) ratio changes significantly in postprandial lipemia. This is not surprising given the smaller amount of TG and CE molecules per particle in HDL compared with the larger LDL and given the increase of HDL2b, which are richer in TG than smaller HDL subfractions. In contrast to HDL, new TGs enter LDL not only via CETP but also via VLDL and IDL entering the LDL density range (37). This pathway is inhibited in postprandial lipemia as CM competes with VLDL for lipases (8), which may be reflected by the significant decrease of ApoB in LDL1 and LDL2. Together with our low number of cases and measurement impression regarding LDL’s TG load, this might explain why neither LDL’s TG/(TG + CE) ratio nor its TG/ApoB ratio (data not shown) do not change significantly in normolipidemics.

Guerin *et al*. (38) reported that the net transfer of CE from HDL to TRL nearly doubles in postprandial lipemia. This fits to our modeled and observed TG net flux data, if an equimolar exchange of CETP is assumed. Our observations and our modeled data (Fig. 4) suggest that considering the total mass of distributed TG via CETP in postprandial lipemia, VLDL remain dominant over CM. This is in line with Lassel *et al*., who report that VLDL1 and not CM is quantitatively the major acceptor of CE redistributed by CETP in postprandial lipemia (39). Thereby, cholesterol-rich pathological TRL remnants are likely derived from VLDL in a prolonged postprandial state, which may exert endothelial damage, for example, in diabetes (40) and pre-eclampsia (41).

CETP, hepatic lipase, and IDL composition and its rate of conversion to LDL are metabolically linked to LDL subfraction distribution. Our model results quantify only the role of CETP. Further, our data suggest that the inhibited IDL-to-LDL conversion affects the buoyant LDL subfraction concentration. Hence, our model may help to entwine how high TG levels lead to a small dense LDL profile. Compared to HDL, the influence of CETP on the redistribution of LDL’s subfraction profile might be a slower one. It might be more dependent on the long-term behavior of postprandial lipemia, namely its duration, frequency, and intensity.

### Discriminating model/model in the postprandial state

When CM and VLDL + CMR are not discriminated, CETP-mediated TG net flux out of TRL and into LDL and HDL is slightly overestimated by 5% and 7%, in the normolipidemic and the HTG samples, respectively. These results suggest that an additional discrimination of the TRL fraction (d > 1.006) into CM and VLDL + CMR is beneficial but not necessary to estimate TG redistribution via CETP into LDL and HDL in postprandial lipemia. From a geometric point of view, the surface of two spheres with identical radii is larger than the corresponding surface of two spheres with the same total volume but different radii. Hence, the surface of TRL is overestimated in the nondiscriminating model where “small” VLDL and “large” CM are lumped together and treated as two spheres with identical radii, whereas in the discriminating model, two spheres with different radii are considered. Since CETP-mediated net flux is calculated via lipoprotein surface, the overestimation of TG net flux is likely because of the overestimation of TRL surface in the nondiscriminating model. Since VLDL remains the dominant particle species and TG source for LDL and HDL in postprandial condition, the additional value of discriminating between VLDL + CMR and CM is not surprisingly small.

Hence, discriminating CM and VLDL + CMR allows us to correct an overestimation of surface and subsequently TG net flux in the postprandial situation of TRL. Our results might enable us to study conditions, in which the postprandial state’s influence on the fasting steady state is increased like the metabolic syndrome or pre-eclampsia. A further interesting application would be, to study how different diets, which are known to influence CM metabolism (42) like n-3 polyunsaturated fatty acids, influence TG redistribution. Studying the genesis of the LDL small dense profile on the basis of longitudinal data would be another interesting application.

Comparing CETP-mediated TG net flux in fasting and postprandial state (Fig. 4), there are two interesting points: *i*) the proportion of TG net flux to HDL and LDL does not change, whereas the net flux increases by >65% and *ii*) although—as expected—the CM fractions share in the CETP-mediated TG redistribution increases massively in postprandial state, the VLDL + CM fraction is still the corresponding driving force, since its surface is in postprandial state still clearly larger compared to the CM surface.

### ApoC3 redistribution

Although there are numerous studies dealing with ApoC3’s kinetics in HDL and VLDL (43, 44), ApoC3’s redistribution in postprandial lipemia has not been statistically analyzed so far. Thus, we here report the observed data for normolipidemics, even though the low number of samples allows no deeper analysis of this issue. As distribution of ApoC3 is likely given by its affinity to different lipoprotein surfaces, redistribution of ApoC3 might be associated to factors leading to a lower affinity of ApoC3 to HDL’s surface. In our study, the normolipidemic postprandial increase of ApoA1 in HDL2b has the highest exploratory power for the redistribution of ApoC3 from HDL to TRL: “The more ApoA1 in HDL2b accumulates postprandial—the more ApoC3 redistributes from HDL to TRL.” As ApoC3 levels are statistically associated with large HDL (14) in the fasting situation, it is in line that the association of redistribution refers to HDL2b, which are the largest HDL subfraction. Likely postprandial-modified HDL2bs, which have a different phenotype than fasting HDL2b (e.g., higher TG/ApoA1 ratios), are poorer acceptors for ApoC3, probably because of altered PL composition or other surface-related factors.

As the focus of this work was on TG, we did not dive deeper into apolipoprotein dynamics.

## Conclusion

Measurement of CM and its components can be performed with a fast and simple approach in a specialized Airfuge® rotation chamber without appropriate isolation of CM.

Our model to estimate CETP-mediated TG redistribution using only the ratios TG/(TG + CE) and surfaces of TRL, IDL, LDL, and HDL (22) is applicable in postprandial lipemia as well—with or without discrimination of TRL into CM and VLDL + CMR fraction (d > 1.006). Discriminating TRL into VLDL + CMR and CM does only improve the TG flux rate estimation by 5% for LDL and 7% for HDL. Thus, postprandial calculations can be done using the classical UC protocol and using the classical “VLDL” fraction as TRL without significant error.

Further, this model allows us to give an individual detailed view on CETPs' influence in the postprandial situation (Fig. 4) based only on the lipoprotein lipid compositions before and after food intake.

In general, VLDL + CMR remain the dominant fraction for CETP-driven TG redistribution even in postprandial state and corresponding direct CM action may be rather overestimated in most researchers’ intuition. Nevertheless, the inhibitory effect of CM on VLDL + CMR metabolism and catabolism leads to significant postprandial changes in lipid distribution among lipoproteins.

The model described in this article may prove to be a valuable tool for studying the impact of imbalanced postprandial lipemia, impaired hydrolysis of TG or metabolic adaptions in pregnancy on LDL and HDL composition, and CETP’s role in atherosclerosis and other metabolic disorders.

## Data availability

Data are listed in the supplemental data.

## Supplemental data

This article contains supplemental data.

## Conflict of interest

The authors declare that they have no conflicts of interest with the contents of this article.

## Figures and Tables

**Supplemental Figure S2:**